# PAN-CANADIAN ESTIMATES OF THE PREVALENCE ASSOCIATED WITH CRITICAL WANDERING AMONG HOME CARE CLIENTS

**DOI:** 10.1093/geroni/igae098.3291

**Published:** 2024-12-31

**Authors:** Antonio Miguel-Cruz, Hector Perez, Micaela Jantzi, Lili Liu, John P Hirdes

**Affiliations:** University of Alberta, Edmonton, Alberta, Canada; University of Waterloo, Waterloo, Ontario, Canada; University of Waterloo, Waterloo, Ontario, Canada; University of Waterloo, Waterloo, Ontario, Canada; University of Waterloo, Waterloo, Ontario, Canada

## Abstract

Wandering is a behaviour common in people living with dementia. Persons living with dementia are at increased risk of getting lost and going missing due to critical wandering. However, the evidence is inconclusive. We used 17 years of cross-sectional clinical assessment records from home care services based on interRAI Home Care assessments to provide Pan-Canadian estimates of the prevalence and clinical risks associated with recent critical wandering. (n=1,598,191). The main clinical variable of interest was recent occurrence of critical wandering. We tested associations between wandering and dementia and cognitive impairment. In all the study regions combined, the overall rate of recent wandering is 3.0%, representing 48,641 unique individuals. The sample was comprised of mainly older adults, with 84.3% over the age of 65. The sample was also predominately female (60.9%). A dementia diagnosis including Alzheimer’s disease or other dementias was present in 23.6% of the sample. Dementia was strongly associated with the prevalence of recent wandering Chi-squared(1, N=1,598,191) = 101,298.866, p < 0.0001). Cognitive impairment was also strongly associated with the prevalence of recent wandering Chi-squared(1, N=1,598,191) = 155,414.738, p < 0.0001). Our analyses also show that persons with wandering behaviour experience many different additional clinical problems that can pose important risks to their health and would require attention of first responders and health professionals. interRAI HC offers insights into wandering risk and health profiles of home care client. This information should be used to manage risk in the community and should be shared with first responders where appropriate.

